# Dysregulated levels of proangiogenic proteins in the placentas of children with autism spectrum disorder and attention-deficit hyperactivity disorder

**DOI:** 10.3389/fmed.2025.1693975

**Published:** 2025-11-27

**Authors:** Cristian Celis, Felipe Troncoso, Eduardo López, Esthefanny Escudero-Guevara, Jesenia Acurio, Carlos Escudero

**Affiliations:** 1 Vascular Physiology Laboratory, Department of Basic Science, Faculty of Sciences, Universidad del Bio Bio, Chillan, Chile; 2 ABCfonoaudiología Therapy Center for Autism Spectrum Disorder, Santiago, Chile; 3 Neurovascular Research and Innovation Consortium, NEUROVAS, Chillan, Chile; 4 Neurology Department, Hospital Clinico Herminda Martin, Chillan, Chile; 5 Faculty of Medicine, Universidad Católica de la Santísima Concepción, Concepción, Chile; 6 Biomedical Sciences Doctorate Program, Universidad de Talca, Talca, Chile; 7 Group of Research and Innovation in Vascular Health (GRIVAS Health), Chillán, Chile

**Keywords:** placenta, VEGF family, autism spectrum disorder, attention-deficit/hyperactivity disorder, angiogenesis

## Abstract

Placental vascularization may influence fetal brain development, but its long-term impact on neurodevelopment remains unclear. In this pilot study, we analyzed two ligand proteins: vascular endothelial growth factor (VEGF) and placental growth factor (PLGF), and their receptors mRNA and protein levels in placentas from children who later were diagnosed with autism spectrum disorder (ASD), attention-deficit/hyperactivity disorder (ADHD), or controls. ASD placentas showed lower VEGF, PLGF, and KDR protein levels but higher FLT1, while ADHD placentas had increased FLT1 and reduced VEGF mRNA. These findings suggest distinct placental vascular alterations in ASD and ADHD, highlighting a potential role of the placenta-brain axis in neurodevelopmental disorders and early-life mechanisms underlying impaired brain development.

## Introduction

Autism spectrum disorder (ASD) and attention-deficit/hyperactivity disorder (ADHD) are common neurodevelopmental conditions with complex etiologies. According to the Diagnostic and Statistical Manual of Mental Disorders, ASD is characterized by social communication and interaction difficulties, along with repetitive behaviors. At the same time, ADHD presents as persistent inattention and/or hyperactivity-impulsivity. Both disorders emerge in early childhood and impact cognitive, social, and emotional development (1).

The reported prevalence of ASD varies across studies and regions. In Chile, the ASD prevalence has been estimated at 1.96% (2021), derived from a cohort of 272 children (2). However, meta-analytic estimates suggest global ASD prevalence in children closer to ∼0.7–1.5%, with substantial heterogeneity across studies and regions (3). Furthermore, global estimations from meta-analyses indicate that ADHD affects approximately 5–8% of children and adolescents, with higher estimates in boys than girls (4, 5), highlighting the need for deeper investigation into prenatal and perinatal contributors.

The etiology of ASD and ADHD involves genetic, epigenetic, and environmental factors, with gene-environment interactions playing a significant role. Prenatal risk factors for ASD include advanced parental age, maternal conditions like gestational hypertension and diabetes, and obstetric complications such as preeclampsia and preterm birth (6). ADHD is highly heritable, but environmental exposures during pregnancy can modulate genetic susceptibility (7). The placental function may play a role in the early programming of these disorders (8). For instance, placental insufficiency and maternal vascular malperfusion, indicative of prenatal hypoxia and nutrient deprivation, have been linked to a markedly increased risk of ASD (9, 10). Also, altered placental DNA methylation patterns were associated with ASD susceptibility (11, 12).

The placenta is a key regulator of fetal development, facilitating the exchange of nutrients and gases, and producing bioactive molecules essential for brain development. Angiogenic factors (ligands), such as vascular endothelial growth factor (VEGF) and placental growth factor (PLGF), as well as their receptors—type 1 (VEGFR-1, FLT1) and type 2 (VEGFR-2, KDR)—play critical roles in placental vascularization (13). Dysregulation of these pathways has been implicated in pregnancy complications, but their potential contribution to neurodevelopmental disorders remains unclear.

Using our placental biobank, we investigated the expression (mRNA and protein) of VEGF, PLGF, FLT1, and KDR in the placentas of children (10–12 years old) who were diagnosed with ASD or ADHD, compared to age-matched controls. This study provides insights into the prenatal origins of these disorders and highlights potential underlying alterations occurring during pregnancy.

## Methods

### Patients

#### Ethical approval and participant recruitment

The Bioethics Committee of the Herminda Martin Hospital in Chillán approved this study. Informed consent was obtained from all parents, and the children also provided informed assent to participate. This pilot study utilized a database from the Vascular Physiology Laboratory at the University of Bio Bio, containing clinical data from 617 deliveries and stored placentas (*n* = 363). Mothers were contacted when their children were 10–12 years old, and a telephone interview was conducted to identify potential participants searching for children with a previous diagnosis of ASD or ADHD.

#### Clinical and neurological assessment

In-person evaluations were scheduled for children with a reported diagnosis of ASD or ADHD. Before this evaluation, we obtained a written informed consent, and a speech-language pathologist (C. Celis) conducted an initial interview to verify the diagnosis. Children were then referred to a neurologist (Dr. E. López) for diagnostic reconfirmation.

#### Final sample and data collection

The final sample consisted of 16 children: 4 with ADHD, 4 with ASD, and eight controls without neurocognitive disorders (Non-ND). Controls were matched by gestational age, pregnancy conditions, child sex, newborn anthropometry, and placental weight. Clinical information was stored in a database, and structured questionnaires were used to collect missing pregnancy-related data.

#### PCR quantitative (qRTPCR)

Total RNA was isolated from placental extracts, and cDNA was synthesized. qRTPCR was performed using specific primers (Supplementary Table 1) for the genes of interest, with gene expression quantified using the ^2–Δ/Δ^CT method (14).

#### Western blot

Placental protein extracts (50 μg) were separated by SDS-polyacrylamide gel electrophoresis and analyzed with primary antibodies anti-VEGF (Santa Cruz, California, OR, USA; sc-7269, 1:1000 dilution), anti-PLGF (Santa Cruz, California, OR, USA; sc-518003, 1:1000), anti-FLT1 (Santa Cruz, California, OR, USA; sc-316, 1:1000), and anti- KDR (Cell Signaling Technology, Denver, MA, USA; 2472, 1:1000 dilution). They were applied overnight independently, followed by incubation with the secondary antibodies Anti-Rabbit IgG (sc-A9169) or Anti-Mouse IgG (sc-A9917) (Sigma-Aldrich, MO, USA). Proteins were normalized with β-actin (Sigma-Aldrich, California, OR, USA; A5441, 1:5000 dilution). The bands were quantified using ImageJ software as previously described (15).

### Statistical analysis

Quantitative variables were presented as mean ± SD, and qualitative variables as percentages. Comparisons between groups were performed using the Kruskal-Wallis test, with Dunn’s *post hoc* test for pairwise comparisons. A *p*-value < 0.05 was considered statistically significant. Data were organized in a Microsoft Excel database, and statistical analyses were conducted using GraphPad Prism.

## Results

### Participant selection and group characteristics

**FIGURE 1:**
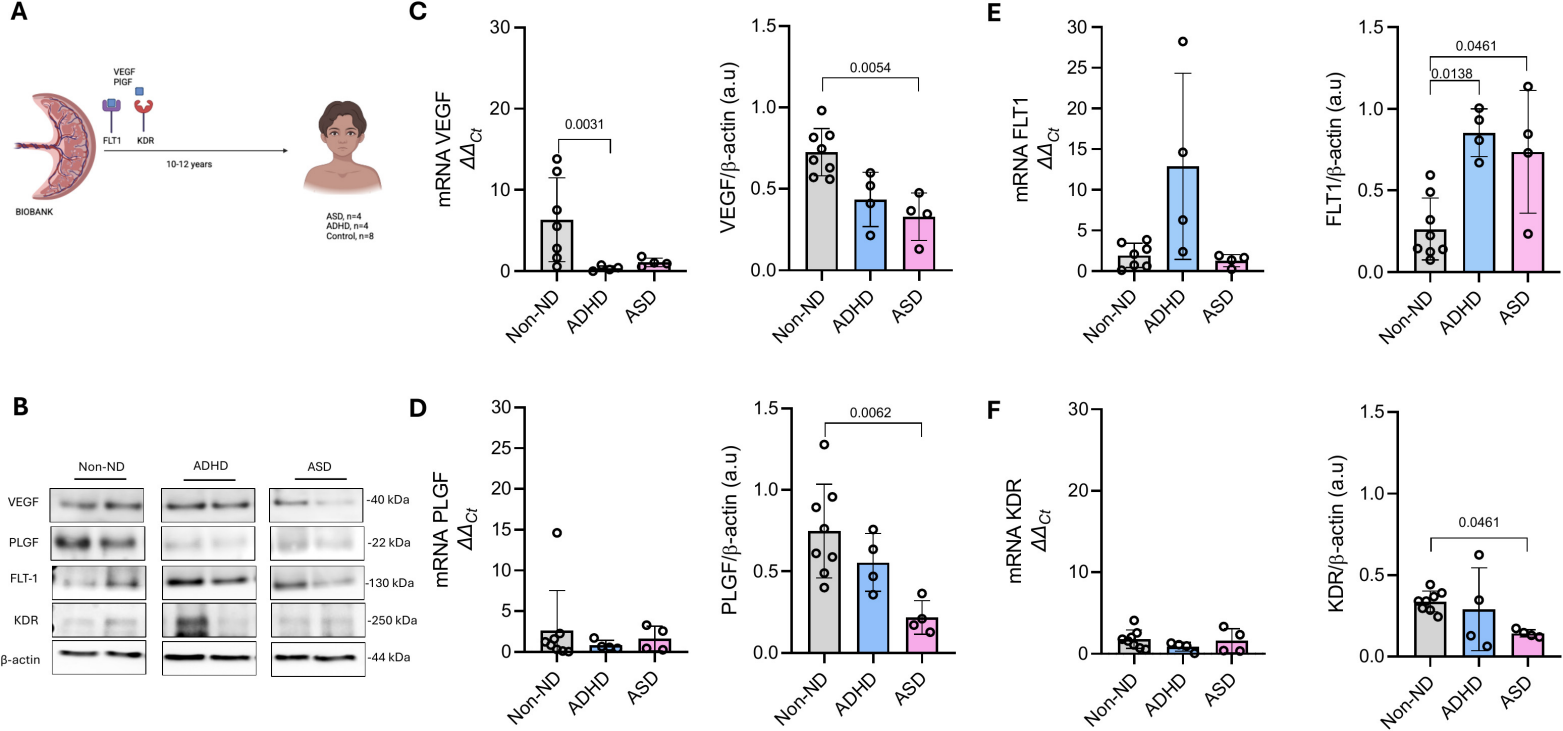
Placental expression of VEGF, PLGF, FLT1, and KDR in children later diagnosed with autism spectrum disorder (ASD), attention-deficit/hyperactivity disorder (ADHD), or controls (Non-ND). **(A)** Schematic representation of the study design, analyzing placental samples stored in our biobank for 10–12 years. Figure A created with Biorender. **(B)** Representative blots of VEGF, PLGF, FLT1, KDR, and β-actin. Protein and mRNA expression of **(C)** VEGF, **(D)** PLGF, **(E)** FLT1, and **(F)** KDR. Every dot represents an individual subject. Data are presented as mean ± SD. *P*-values are included in each graph.

Of the 617 potential participants, only 363 had placenta samples in our database. From then, 212 mothers were contacted, and 24 agreed to participate (Supplementary Figure 1). The final sample included in this pilot study consists of four placentas and children with ADHD, four with ASD, and eight with non-neurocognitive disorders (Non-ND) (Figure 1A). The age range at inclusion was 10–12 years, with no significant differences among groups. A Kruskal–Wallis test indicated significant differences among groups in mothers’ age [H(2) = 8.53, *p* = 0.0053, *n* = 16]. *Post hoc* Dunn’s test showed a significant difference in the age of mothers of children with ADHD compared to those of the Non-ND group (*Z* = 2.92, *p* = 0.0106). No significant differences were observed in gestational age, newborn anthropometry, or placental weight (Table 1).

**TABLE 1:** Characteristics of the included children.

Analyzed characteristic	Non-ND	ADHD	ASD	p
N	8	4	4	0.753
Children’s age (years)	12.8 ± 0.3	12.5 ± 0.5	12.7 ± 0.5	
Maternal age at pregnancy (years)	**33.8 ± 5.8**	19.25 ± 3.3*	**30.25 ± 7.4**	**0.0053**
Primipara (*n*, %)	2 (25)	3 (75)	2 (50)	
Gestational age (weeks)	38.3 ± 1.2	36.3 ± 4.6	38.3 ± 0.9	0.697
Pregnancy complications (*n*, %)	5 (62.5)	3 (75)	4 (100)	–
Cesarean section (*n*, %)	4 (50)	1 (25)	4 (100)	–
Newborn sex (male/female)	5/3	2/2	1/3	–
Weight (gr)	3537 ± 642.3	2535 ± 744.0	3793 ± 798.2	0.055
Size (cm)	49.4 ± 1.6	45.0 ± 4.8	48.5 ± 4.0	0.109
Placental weight (gr)	517.5 ± 90.4	495 ± 137	580 ± 181	0.496
Placental efficiency (gr/gr)	**0.14 ± 0.02**	0.19 ± 0.01*	**0.15 ± 0.03**	**0.006**

Non-ND, non-neurocognitive disorders. Any pregnancy complications, including podalic presentation, preeclampsia, gestational diabetes, and preterm delivery.

*p < 0.05 versus Non-ND. Bold represents statistical differences. Kruskal-Wallis test followed by Dun’s multiple comparisons test.

Placental efficiency was calculated (grams of fetal mass per gram of placental mass), a widely used measure of how much fetal mass is produced per gram of placental mass. This ratio had shown variations associated with pregnancy outcomes (16). We found that placental efficiency was significantly different among the studied groups [H(2) = 8.49, *p* = 0.0061], with higher values in the ADHD group than in the Non-ND group (*Z* = 2.79, *p* = 0.016).

Pregnancy complications tended to be more frequent among mothers of children with ADHD (3/4) and ASD (4/4) compared to those of children in the Non-ND group (5/8). Among these complications, preeclampsia was reported in 2/4 ADHD cases, 3/4 ASD cases, and 2/8 in the Non-ND group. Additionally, gestational diabetes was observed in two mothers from the Non-ND group and one from the ASD group. Only one case of preterm delivery was reported, occurring in the ADHD group.

### Placental protein and gene expression analysis

Figure 1B shows representative blots of analyzed proteins. A Kruskal–Wallis test indicated significant differences among groups in the placental protein levels of VEGF [H(2) = 10.48, *p* = 0.0006]; PLGF [H(2) = 8.75, *p* = 0.0038]; FLT1 [H(2) = 9.41, *p* = 0.0019]; and KDR [H(2) = 5.22, *p* = 0.050]. Placentas from children with ASD exhibited significantly lower protein levels of VEGF (Figure 1C, *Z* = 3.00, *p* = 0.0054), PLGF (Figure 1D, Z = 2.96, *p* = 0.0062), and KDR (Figure 1F, *Z* = 2.27, *p* = 0.046) compared to children in the Non-ND group. These reductions were not reflected at the mRNA level. Conversely, compared to children in the Non-ND group, FLT1 protein levels were significantly higher in the ASD group (Figure 1E, *Z* = 2.27, *p* = 0.046) despite no differences in FLT1 mRNA expression.

Placentas of children with ADHD showed significantly lower VEGF mRNA levels (Figure 1C, *Z* = 2.95, *p* = 0.0031) and elevated FLT1 protein levels (Figure 1F, *Z* = 2.70, *p* = 0.013) compared to children in the Non-ND group. No significant differences were observed in VEGF, PLGF, or KDR protein levels.

There were no statistically significant differences in any of the analyzed markers between the ASD and ADHD groups.

## Discussion

The results indicate that ASD is associated with a deficiency in angiogenic agonists (VEGF and PLGF) and their receptor, KDR, as well as an increase in FLT1 in the placenta. ADHD placentas exhibit a distinct angiogenic imbalance with elevated FLT1 protein level. These findings suggest potential early-life placental vascular disruptions that may contribute to the intrauterine initiation of altered neurodevelopmental trajectories.

A growing body of evidence supports the role of the placenta in brain development and the etiology of neurodevelopmental disorders (8, 9, 17). Studies have linked placental abnormalities to an increased risk of ASD and ADHD (11, 12, 18). For instance, preeclampsia, a condition characterized by impaired placental vascularization (15), has been associated with an increased risk of ASD and developmental delay (19). For ADHD, placental stress responses and angiogenic imbalances may also contribute to its pathogenesis (20). Compatible with this finding, placentas in the ADHD group showed an increase in insufficiency compared with the control group. Moreover, epidemiologic studies suggest an increased risk of neurodevelopmental diagnoses after abruption of the placenta, though specificity for ASD was not directly analyzed (21).

Placental insufficiency and maternal vascular malperfusion have been observed in ASD cases, suggesting that prenatal hypoxia and nutrient deprivation may contribute to altered brain development (9, 10). For instance, medical record analysis showed that acute signs of vascular placental alterations (chronic uteroplacental vasculitis or maternal vascular malperfusion) were highly associated with risk of ASD (7 to 12-fold higher risk) (9). Placental trophoblast inclusions, a marker of altered placental development, have been reported at significantly higher rates in ASD cases than in controls, suggesting that structural abnormalities in the placenta may be early indicators of neurodevelopmental risk (22, 23). Additionally, epigenetic modifications in the placenta, including DNA methylation changes at key neurodevelopmental genes, have been identified in ASD cases, further supporting the placenta’s role in fetal brain programming (11, 12). These observations are consistent with the possibility that acute placental injury or chronic placental dysfunction might perturb angiogenic signaling and fetal neurovascular development, but prospective mechanistic evidence is lacking.

Our findings align with this literature, as reduced levels of critical proangiogenic proteins (such as VEGF and PLGF) in ASD placentas may indicate compromised placental vascularization, potentially leading to fetal brain hypoxia, which in turn may have long-lasting consequences. Future studies that include CD31 immunohistochemistry and stereological/morphometric analysis are required to determine whether vessel density or architecture is altered in these placentas and how these alterations may predispose to structural/functional changes.

In this regard, our findings of lower VEGF/PLGF and KDR protein with higher FLT1 in ASD placentas (and increased FLT1 in ADHD placentas) are consistent with preclinical evidence linking placental angiogenic signaling to fetal brain vascular development. For example, Lecuyer et al. showed that prenatal alcohol exposure impaired placental angiogenesis, reduced PLGF levels, and altered fetal brain vasculature. Interestingly, placental repression of PLGF altered brain FLT1 expression and mimicked alcohol-induced vascular defects in the cortex. At the same time, overexpression of placental PLGF rescued alcohol effects on fetal brain vessels. Translational evidence in humans showed that alcohol exposure disrupted both placental and brain angiogenesis (24). Supporting this idea, another report showed that repression of placental CD146, a co-receptor of KDR, led to reduced cortical vessel density and oligodendrocyte loss (25). Moreover, placental Insulin-like Growth Factor 1 (IGF1) has been shown to induce persistent neurodevelopmental changes in striatal development (26). These preclinical studies provide mechanistic support for a placenta: brain axis by which altered placental angiogenic signaling may affect fetal neurovascular development and, potentially, later cognitive outcomes. Despite that, we acknowledge that our human data are exploratory and do not demonstrate causality.

Considering embryonic development, Manzo et al. (27) emphasize the importance of neural tube vascular events occurring during early embryogenesis. We note that analyses of term placentas provide a window into cumulative or persistent placental changes but cannot directly demonstrate that the specific angiogenic disruptions we measured were present during neural tube closure. Term placental measures may function as surrogate or residual markers of earlier placental dysfunction, but prospective sampling in early pregnancy is required to test temporality and causality.

The pattern of elevated FLT1 with reduced VEGF and PlGF could create a functionally anti-angiogenic environment by sequestering free ligand, analogous to mechanisms implicated in preeclampsia (28). Alternatively, FLT1 upregulation might reflect an adaptive placental response to chronic stressors (hypoxia or inflammation). Our current data (term placenta protein quantification) cannot distinguish these possibilities. We encourage future work to enhance our understanding of how this imbalance between ligands (VEGF/PLGF) and the FLT1 receptor may drive placental vascular alterations that may impair brain vascular function.

We acknowledge that this is a small, exploratory pilot study, for which analyses are underpowered for sex-stratified comparisons and covariate adjustment. These results should therefore be considered hypothesis-generating. Nevertheless, the study used precious placental samples stored for over a decade, providing a rare opportunity to analyze long-term biological markers. The retrospective nature of the study limits causal inference, and future prospective studies with larger cohorts are needed to validate these findings. Despite that, by integrating protein and mRNA analyses, we identified differential regulatory patterns in placentas from individuals with ASD and ADHD, providing novel insights into early-life vascular alterations that may influence neurodevelopment. Additionally, our findings contribute to the growing field of the placenta-brain vascular axis (17).

## Conclusion

In conclusion, this study highlights distinct placental angiogenic profiles in ASD and ADHD, suggesting that early-life vascular imbalances may contribute to neurodevelopmental disorders. Further studies are needed to confirm these associations and explore potential interventions to improve placental vascular health.

## Data Availability

The raw data supporting the conclusions of this article will be made available by the authors, upon request.
