# Supplementary material for: Dysregulated levels of proangiogenic proteins in the placentas of children with autism spectrum disorder and attention-deficit hyperactivity disorder
Source: Front Med (Lausanne). 2025 Nov 27;12:1693975. doi: 10.3389/fmed.2025.1693975 (PMC12697063; doi:10.3389/fmed.2025.1693975)
Supplement: Supplementary file 1 [file Data_Sheet_1.docx]

**Dysregulated levels of proangiogenic proteins in the placentas of children with autism spectrum disorder and attention-deficit hyperactivity disorder**

Cristian Celis^1,2‡^; Felipe Troncoso^1,3‡^; Eduardo López^3,4,5^; Esthefanny Escudero-Guevara^1,6^ Jesenia Acurio^1^; Carlos Escudero^1, 3,7*^.

^1^ Vascular Physiology Laboratory. Department of Basic Science, Faculty of Sciences. Universidad del Bio Bio, Chillan, Chile.

^2^ ABCfonoaudiología Therapy Center for Autism Spectrum Disorder, Santiago, Chile

^3^ Neurovascular Research and Innovation Consortium, NEUROVAS, Chillan, Chile

^4^ Neurology Department, Hospital Clinico Herminda Martin, Chillan, Chile

^5^ Faculty of Medicine, Universidad Católica de la Santísima Concepción, Concepción, Chile

^6^ Biomedical Sciences Doctorate Program, Universidad de Talca, Talca, Chile.

^7^ Group of Research and Innovation in Vascular Health (GRIVAS Health), Chillán, Chile.

Short title: ASD, ADHD, and placenta angiogenesis

‡ These authors have made similar contributions to this manuscript

*Correspondence:

Carlos Escudero, MD, PhD

Vascular Physiology Laboratory,

Group of Research and Innovation in Vascular Health (GRIVAS Health)

Basic Sciences Department

Faculty of Sciences

Universidad del Bio-Bio

Chillán, Chile

Phone: 56-42-2463019 / Mobile: 56-9-65655127

cescudero@ubiobio.cl

ORCID: 0000-0001-7688-4621

Word count: 3944

Conflict of interest: none

Number of Figures: 1

Number of Tables: 1

Supplementary information: yes

Number of references: 28

**Table S1. Primers used during QPCR analysis**

| Gen | Gen-Bank Oligonucleotides | | Tm (°C) | | Amplicon (pb) | |
| --- | --- | --- | --- | --- | --- | --- |
| KDR | NM_002253.4 | F 5”- CTT CGA AGC ATC AGC ATA AGA AAC T-3´  R 5”- TGG TCA TCA GCC CAG TGG A -3 | 53 | 156 | |  |
| VEGF | AF024710.1 | F 5´- CTT GCC TTG CTG CTC TAC CT -3´  R5′- GGC-TGG-GGT-GTT-GAA-GGT-3´ | 56 | 325 | |  |
| PLGF | NM_002632.6 | F 5´- CGG CTC GTC AGA GGT GGA AG -3´  R5′- GCA GGG AGA CAC AGG ATG GG -3´ | 57 | 143 | |  |
| FLT1 | AF063657.2 | F-3´TCC CTT ATG ATG CCA GCA AGT -3´  R5”-CCA AAA GCC CCT CTT CCA A -3´ | 53 | 79 | |  |
| GAPDH | NM_001357943.2 | F 5′- ACA GTC AGC CGC ATC TTC TTT -3´  R 5′- GGC GCC CAA TAC GAC CAA AT -3´ | 55 | 104 | |  |

Oligonucleotides for humans are synthesized with the PrimerBlast tool (NCBI) by aligning the complete mRNA sequence for each gene. Tm values ​​were analyzed with the AmplifX® Gen-Bank program AmplifX® Gen-Bank.

**Figure S1. Flowchart of participant inclusion in the study.** The database included 617 individuals, of whom 363 had available samples. Of these, 212 were contacted, while 151 could not be reached. Among those contacted, 24 agreed to participate and were categorized into controls (n=16), ADSD (n=4), and ASD (n=4). The majority (188) declined participation due to various reasons, including incorrect phone numbers (n=77), lack of response (n=37), and other factors (n=72).
